# Polyunsaturated fatty acids in serum and homocysteine concentrations in Japanese men and women: a cross-sectional study

**DOI:** 10.1186/1743-7075-10-41

**Published:** 2013-06-10

**Authors:** Ayami Kume, Kayo Kurotani, Masao Sato, Yuko Ejima, Ngoc Minh Pham, Akiko Nanri, Keisuke Kuwahara, Tetsuya Mizoue

**Affiliations:** 1 Department of Epidemiology and Prevention, Clinical Research Centre, National Centre for Global Health and Medicine, 1-21-1 Toyama, Shinjuku-ku, Tokyo 162-8655, Japan; 2 Laboratory of Nutrition Chemistry, Faculty of Agriculture, Kyushu University, 6-10-1 Hakozaki, Fukuoka, Higashi-ku, Japan

**Keywords:** Cholesterol ester, Cross-sectional studies, Docosahexaenoic acid, Fatty acids, Homocysteine, Polyunsaturated fatty acids, Phospholipids

## Abstract

**Background:**

Supplementation studies have suggested a role of n-3 polyunsaturated fatty acids (PUFAs) in homocysteine metabolism, but the evidence is limited and inconsistent among studies that measured blood levels of n-3 and n-6 PUFAs. We examined the association between blood levels of PUFAs and homocysteine in Japanese men and women.

**Methods:**

The subjects were 496 employees (290 men and 206 women) of 2 municipal offices in Japan. Fatty acid composition in serum phospholipids and cholesterol ester (CE) was measured using gas–liquid chromatography. Multiple regression was used to calculate means of homocysteine concentrations according to PUFA tertile with adjustment for potential confounders.

**Results:**

Serum homocysteine concentration decreased with increasing levels of total n-3 PUFA, eicosapentaenoic acid and docosahexaenoic acid (DHA) in serum phospholipids and CE with adjustment for age, sex and workplace. However, only DHA in serum phospholipids remained statistically significant after additional adjustment for other potential confounders including serum folate (P-trend = 0.04). N-6 PUFAs were not significantly associated with homocysteine concentrations.

**Conclusions:**

Higher proportion of DHA in serum phospholipids may be associated with lower homocysteine concentrations in Japanese men and women.

## Background

Circulating total homocysteine is a well-known factor associated with risk of cardiovascular disease [1,2], although the reduction of homocysteine levels using folic acid supplementation has no effect on vascular outcomes [3]. It is a sulfur amino acid, which is an intermediate in the lineal pathway of synthesis of cysteine from methionine [4]. Homocysteine metabolism stands at the intersection of two pathways: remethylation to methionine, which requires folate and vitamin B12, and transsulfuration to cystathionine, which requires vitamin B6 [5]. Consequently, deficiencies of vitamin B6, B12 and folate cause elevations of blood homocysteine [6].

Evidence has been accumulating that supports a protective role of n-3 polyunsaturated fatty acids (PUFAs) against coronary heart disease (CHD) [7,8], although recent meta-analyses show no or insufficient evidence regarding the effect of n-3 PUFA supplementation on major cardiovascular events in high risk populations [9,10]. N-3 PUFA may decrease CHD risk through various mechanisms; they prevent cardiac arrhythmia, decrease serum triglyceride and cholesterol levels, reduce platelet aggregation, and lower inflammation [11]. Besides, a recent experimental study showed that n-3 PUFAs at super-physiological levels regulate mRNA expression of the genes encoding the key enzymes involved in homocysteine metabolism [12]. In addition, a meta-analysis of intervention studies suggests that n-3 PUFA supplementation can decrease the plasma homocysteine concentrations [13]. In an observational study, higher dietary intake of n-3 PUFAs was associated with decreased plasma homocysteine concentrations [14]. While fat intake estimated from dietary questionnaire tends to be underestimated [15], fatty acid composition in cholesterol ester (CE) and phospholipids is a good biomarker of habitual dietary intake of fatty acid [16].

To date, several studies reported an inverse association between docosahexaenoic acid (DHA) in phospholipids [17-20] and erythrocyte [21] and homocysteine, whereas such association has not been reported for eicosapentaenoic acid (EPA) [18-20] and alpha-linolenic acid (ALA) [19,20]. The findings of these studies, however, may be limited due to a lack of adjustment for folate [18,20,21], vitamin B6 [17-21], vitamin B12 [18,20,21], physical activity [17-21] and smoking status [18-21], all of which are important determinants of homocysteine concentrations [22]. As regards the association between homocysteine and n-6 PUFAs, which have also been linked to a lower risk for cardiovascular disease if supplemented in combination with n-3 PUFAs [23], a few studies with blood fatty acid measurement showed inconsistent results [19,20,24].

Epidemiologic data on this issue so far have been derived from populations with low n-3 PUFA intakes, and the evidence is lacking in a population with high n-3 PUFA levels. Fish consumption in Japan is among the highest in the world [25] and Japanese have much higher blood concentrations of long-chain n-3 PUFAs as well as lower prevalence of atherosclerotic changes compared with white Americans [26]. Here, we investigate the associations between PUFAs in serum CE and phospholipids and serum homocysteine among a healthy Japanese population who have high PUFA intakes.

## Methods

### Subjects

The present cross-sectional study was based on a health survey conducted in July and November 2006 among employees of 2 municipal offices in north-eastern Kyushu, Japan. All full-time employees except for those on long sick leave or maternity leave were invited to participate in the survey (n = 601), during which researchers obtained data on anthropometric measurements, collected venous blood and urine specimens, and inquired about lifestyle via questionnaire. A total of 547 subjects participated (323 men and 224 women, aged 21–67 y), which gave a response rate of 91%. We excluded subjects with a history of cancer, diabetes, or cardiovascular disease or who were receiving medication for hyperlipidemia, as well as those with missing information on serum homocysteine, serum fatty acids, and covariates, leaving a total of 496 Japanese subjects (290 men and 206 women) for analysis. The protocol of the study was approved by the Ethics Committee of the National Centre for Global Health and Medicine and written informed consent was obtained from all subjects.

### Serum sampling

Participants were instructed to receive the check-up after an overnight fast. A total of 7 mL venous blood was drawn into a vacuum tube and then conveyed to a laboratory in a cooler box. The blood was centrifuged for 15 min and the separated serum was divided into a maximum of 6 tubes (0.5 mL each). Five of these tubes were stored at −80°C or at −20°C (one tube for the measurement of fatty acid composition) until analysis.

### Measurement of serum fatty acid composition

After serum lipids were extracted by the Folch method [27], CE and phospholipids were separated by thin-layer chromatography on silica gel G. The plates containing the serum lipid extracts were developed with petroleum ether/dietylether/acetic acid (82:18:1, vol/vol/vol). Fatty acids liberated from CE and phospholipids were stripped from areas at 1.0 Rf value and spotting origin, respectively, on the silica gel plate (25 TLC plate 20 × 20 cm, Silica gel 60 purchased from MERCK, Darmstadt, Germany). CE and phospholipids absorbed in the silica gel powders were methylated with sulphuric acid/methanol (1:115, vol/vol) solution and the methyl esters in the solution were extracted with hexane 3 times. The hexane was dried up under nitrogen gas and then the fatty acid methyl esters in a small amount (less than 20 μL) of hexane were applied to gas chromatography with OMEGA WAX 320 FUSED SILICA Capillary Column (30 m long, 0.32 mm i.d., 0.25 mm film thickness) obtained from SUPELCO (Bellefonte, PA, USA). Methyl esters of fatty acids on the gas-chromatogram were identified using a commercially available standard of Grain Fatty Acid Methyl Ester Mix (SUPELCO). The methyl esters of γ-linolenic acid, dihomo-γ-linolenic acid, EPA and DHA, which were not included in the standard mix, were identified by using methyl ester standard for each of these fatty acids (SUPELCO). The fatty acid methyl ester values were calculated as a weight percentage based on each peak area. The coefficient of intra-assay variation values for major fatty acid methyl esters were as follows: 18:2 n-6 (0.5%), 18:3 n-6 (6.3%), 20:3 n-6 (4.6%), 20:4 n-6 (4.3%), 20:5 n-3 (5.7%) and 22:6 n-3 (5.6%) for CE; 16:0 (4.0%), 18:0 (1.6%), 16:1 (8.1%), 18:1 (4.9%), 18:2 n-6 (1.0%), 20:3 n-6 (2.8%), 20:4 n-6 (3.5%), 20:5 n-3 (3.5%), 22:5n-3 (4.8%) and 22:6 n-3 (10.0%) for phospholipid.

### Measurement of serum folate and homocysteine

Serum homocysteine concentrations were measured using HPLC with a Shimadzu LC 9A system. The coefficient of variation values for homocysteine measurement was 2.1% at 8.5 nmol/mL. Serum folate concentrations were measured using chemiluminescent immunoassay with Chemilumi-ACS folate II kit (Siemens Healthcare Diagnostics Co. Ltd., Japan). All these measurements were conducted at an external laboratory (Mitsubishi Chemical Medience, Tokyo, Japan).

### Dietary assessment

Dietary habit during the preceding month was assessed using a validated brief self-administered diet history questionnaire (BDHQ), which consists of five sections: 1) the frequency of 46 food and non-alcoholic beverage intake; 2) daily frequency of rice and miso soup intake; 3) the frequency of alcoholic drinking and the amount of consumption for five alcoholic beverages per typical drinking occasion; 4) usual cooking method; and 5) dietary behaviour. Dietary intakes for 58 food and beverage items, energy and selected nutrients were estimated using an ad hoc computer algorithm for the BDHQ, with reference to the standard tables of food composition in Japan [28]. According to the validation study of the BDHQ using 16-day weighted dietary records as the gold standard, Pearson correlation coefficients for energy-adjusted intake of vitamin B6 and vitamin B12 were 0.43 and 0.34, in men, respectively and 0.48 and 0.30, in women, respectively [29].

### Lifestyle and anthropometric assessment

Body height was measured to the nearest 0.1 cm with the subject standing without shoes. Body weight in light clothes was measured to the nearest 0.1 kg. Body mass index (BMI) was calculated as body weight in kilograms divided by the square of body height in meters. Occupational physical activity was categorized as either sedentary or active work according to job title; clerical jobs were classified as sedentary work, whereas other types of work, including child-care, cafeteria work and technical jobs were classified as active work. Non-occupational physical activity was expressed as metabolic equivalent hours per week (MET-h/wk) based on the usual frequency and duration of five different activities (walking; low-, moderate- and high-intensity activities; and gardening); data on these activities were collected in the self-reported lifestyle questionnaire. As regards alcohol consumption, the amount of ethanol consumed per day was estimated based on the frequency of alcohol drinking and the amount of alcohol consumed per occasion.

### Statistical analyses

Differences in the proportions and means of potential confounding variables across tertile categories of n-3 PUFA and n-6 PUFA were assessed by using the Mantel-Haenszel chi-square test for categorical variables and linear regression analysis for continuous variables, with ordinal numbers 0–2 assigned to the tertile categories of fatty acids, respectively. The relation between serum fatty acid (tertile) and homocysteine concentration was assessed by using multiple regression. We calculated geometric mean and its 95% confidence intervals (CIs) of homocysteine concentrations (nmol/mL) for each tertile of serum fatty acid. The confounding variables considered were age (y, continuous), sex, workplace [A (surveyed in July) or B (surveyed in November)], BMI (kg/m^2^, continuous), smoking status (non-smoker or current smoker), alcohol consumption (non-drinker, drinker consuming <20 g of ethanol/d, or drinker consuming ≥20 g of ethanol/d), occupational physical activity (sedentary work or active work), non-occupational physical activity (0, 0 < to <5 or ≥5 MET-h/wk), log-transformed serum folate (ng/mL, continuous), vitamin B6 intake (mg/1000 kcal, continuous) and log-transformed vitamin B12 intake (μg/1000 kcal, continuous). The first model was adjusted for age, sex and workplace (model 1); and the second model was further adjusted for BMI, smoking, alcohol consumption, occupational physical activity, non-occupational physical activity, serum folate, vitamin B6 intake and vitamin B12 intake (model 2). We adjusted for serum folate instead of folate intake because in the present population, serum folate had a greater impact on the changes in odds ratio than did dietary folate. We used dietary data on vitamin B6 and vitamin B12 because we have serum B6 data only for a subgroup of the study population and no data on serum vitamin B12 concentrations. There were no statistically significant interactions by sex (p for interaction > 0.1 for all fatty acids), and thus results were presented for men and women combined. Two-sided P values less than 0.05 were regarded as statistically significant. All analyses were performed using STATA version 12.0 (STATA Corp., College Station, TX, USA).

## Results

Characteristics of study subjects according to tertile category of serum n-3 and n-6 PUFA in CE are shown in Table 1. Participants with higher serum n-3 PUFA were older, more likely to be male, smokers and heavy drinkers and had higher levels of BMI, serum folate and dietary vitamin B12 intake, compared with those with lower n-3 PUFA. Participants with higher serum n-6 PUFA levels were younger, less likely to be male, smokers and heavy drinkers, had lower levels of BMI, non-occupational physical activity and dietary vitamin B12 intake, but had higher concentrations of serum folate, compared with those with lower n-6 PUFA. Similar associations were observed when participants were categorized according to serum n-3 and n-6 PUFAs in phospholipids (data not shown).

**Table 1:** **Characteristics of the subjects according to tertile of n**-**3 PUFA and n**-**6 PUFA in serum cholesterol ester**

		**n-3 PUFA**				**n-6 PUFA**		
	**T1 (2.74)**^**1**^	**T2 (4.01)**	**T3 (5.79)**	***P*-trend**^**2**^	**T1 (58.36)**	**T2 (62.58)**	**T3 (66.18)**	***P*-trend**
No. of subjects	166	165	165		166	165	165	
Age (y, mean ± SD)	37.9 ± 9.3	41.8 ± 10.5	47.7 ± 9.8	<0.001	47.9 ± 10.1	41.4 ± 10.4	38.1 ± 8.9	<0.001
Sex (male,%)	52.4	54.6	68.5	<0.001	79.5	57.0	38.8	<0.001
Workplace (A,%)^3^	31.3	31.5	29.7	0.75	29.5	31.5	31.5	0.69
Body Mass Index (kg/m^2^, mean ± SD)	21.9 ± 3.3	22.5 ± 3.9	23.0 ± 3.2	0.01	23.5 ± 3.5	22.3 ± 3.5	21.6 ± 3.2	<0.001
Current smoking (%)	21.1	28.5	32.7	0.02	43.4	26.1	12.7	<0.001
Alcohol consumption (≥20 g of ethanol/d,%)	28.9	40.0	58.2	<0.001	64.5	35.2	27.3	<0.001
Occupational physical activity (sedentary,%)	80.1	77.6	83.6	0.12	81.9	84.2	75.2	0.42
Non-occupational physical activity (≥5 MET-h/wk,%)	27.7	29.7	30.3	0.60	36.8	30.3	20.6	0.001
Serum folate (ng/ml)	3.4 (2.6, 4.9)^4^	3.8 (2.7, 5.0)	4.3 (3.1, 5.8)	0.002	3.4 (2.7, 4.7)	3.8 (2.7, 5.7)	4.0 (3.0, 5.3)	0.005
Dietary intake of vitamin B6								
(mg/1000 kcal, mean ± SD)	0.6 ± 0.2	0.7 ± 0.2	0.7 ± 0.1	0.18	0.6 ± 0.2	0.7 ± 0.2	0.7 ± 0.1	0.28
Dietary intake of vitamin B12 (μg/1000 kcal)	3.9 (2.8, 5.0)	4.1 (3.1, 5.2)	5.1 (4.0, 6.2)	<0.001	4.8 (3.6, 6.1)	4.3 (3.0, 5.9)	4.0 (3.0, 5.0)	<0.001

Abbreviations: *T* Tertile, *PUFA* Polyunsaturated fatty acids, *SD* Standard deviation, *MET* Metabolic equivalent.

^1^Median percentage of n-3 PUFA or n-6 PUFA (all such values).

^2^P-trend values were based on the Mantel-Haenszel chi-square test for categorical variables and on linear regression analysis for continuous variables,with ordinal numbers 0–2 assigned to tertile categories of each fatty acid.

^3^A, workplace surveyed in July.

^4^Median (interquartile range) of serum folate and dietary intake of vitamin B12.

Pearson correlation coefficient was calculated to assess correlation between each PUFA. In phospholipids, relatively strong correlations (absolute value of *r* > 0.6) were observed between DHA and DPA (*r* = 0.71), between LA and EPA (r = −0.60), and between LA and DHA (*r* = −0.60). As regards CE, relatively strong correlations (absolute value of *r* > 0.6) were observed between EPA and DHA (*r* = 0.70), between GLA and DGLA (*r* = 0.64). The correlation coefficients of total PUFA with n-6 PUFA and n-3 PUFA in phospholipids were 0.42 and 0.35, respectively, whereas the corresponding figures in CE were 0.91 and −0.07, respectively. Serum homocysteine concentration showed relatively strong, inverse correlation with serum folate (*r* = −0.53) and moderate, inverse correlation with dietary intake of folate (*r* = −0.30) and vitamin B6 (*r* = −0.28), but not with dietary vitamin B12 (*r* = −0.05).

The relation between serum phospholipids fatty acid composition and homocysteine concentration is shown in Table 2. Homocysteine concentration was significantly lower with increasing levels of total n-3 PUFA, EPA and DHA in age-, sex- and workplace-adjusted model (P-trend = 0.02, 0.01 and 0.003, respectively). In the fully adjusted model, the associations or total n-3 PUFA and EPA were attenuated (P-trend = 0.36 and 0.12, respectively), whereas that for DHA remained statistically significant (P-trend = 0.04). Total n-6 PUFA and its components were not significantly associated with homocysteine concentration in either model.

**Table 2:** **Mean serum homocysteine concentrations according to tertile of n**-**3 PUFA and n**-**6 PUFA in serum phospholipids**

**Fatty acid tertile**	**n**	**Model 1**^**1**^	**Model 2**^**2**^
PUFA			
<44.32%	166	10.58 (10.13, 11.05)^3^	10.45 (10.05, 10.88)
44.32–46.20%	165	10.23 (9.81, 10.68)	10.16 (9.77, 10.55)
≥46.21%	165	9.95 (9.52, 10.39)	10.14 (9.75, 10.55)
*P*-trend^4^		0.06	0.34
n-6 PUFA^5^			
<32.44%	166	10.18 (9.72, 10.66)	10.32 (9.90, 10.77)
32.44–35.19%	165	10.05 (9.63, 10.49)	9.98 (9.61, 10.37)
≥35.20%	165	10.52 (10.05, 11.02)	10.45 (10.02, 10.90)
*P*-trend^4^		0.34	0.67
LA (18:2n-6)			
<19.98%	166	10.32 (9.90, 10.77)	10.08 (9.64, 10.55)
19.98–22.92%	165	9.98 (9.61, 10.37)	10.12 (9.70, 10.56)
≥22.93%	165	10.45 (10.02, 10.90)	10.55 (10.09, 11.04)
*P*-trend^4^		0.67	0.14
DGLA (20:3n-6)			
<1.96%	166	10.11 (9.68, 10.55)	10.25 (9.85, 10.66)
1.96–2.43%	165	10.47 (10.04, 10.93)	10.39 (9.99, 10.79)
≥2.44%	165	10.17 (9.74, 10.62)	10.12 (9.71, 10.54)
*P*-trend^4^		0.83	0.64
AA (20:4n-6)			
< 9.25%	166	10.30 (9.86, 10.75)	10.29 (9.85, 10.65)
9.25 –10.71%	165	10.27 (9.84, 10.72)	10.39 (10.00, 10.80)
≥ 10.72%	165	10.18 (9.75, 10.63)	10.12 (9.73, 10.52)
*P*-trend^4^		0.73	0.64
n-3 PUFA^5^			
<9.95%	166	10.75 (10.29, 11.23)	10.49 (10.07, 10.92)
9.95–12.34%	165	10.08 (9.66, 10.52)	10.07 (9.69, 10.46)
≥12.35%	165	9.93 (9.50, 10.38)	10.20 (9.79, 10.62)
*P*-trend^4^		0.02	0.36
EPA (20:5n-3)			
<1.87%	166	10.76 (10.31, 11.24)	10.56 (10.14, 10.99)
1.87–2.89%	165	10.10 (9.68, 10.54)	10.12 (9.74, 10.51)
≥2.90%	165	9.90 (9.48, 10.34)	10.08 (9.69, 10.49)
*P*-trend^4^		0.01	0.12
DPA (22:5n-3)			
<0.99%	166	10.61 (10.15, 11.09)	10.49 (10.08, 10.92)
0.99–1.20%	165	10.14 (9.72, 10.58)	10.09 (9.71, 10.48)
≥1.21%	165	10.01 (9.57, 10.48)	10.18 (9.76, 10.60)
*P*-trend^4^		0.08	0.30
DHA (22:6n-3)			
<6.84%	166	10.72 (10.26, 11.19)	10.57 (10.16, 11.00)
6.84–8.25%	165	10.33 (9.90, 10.77)	10.25 (9.87, 10.64)
≥8.26%	165	9.73 (9.31, 10.17)	9.94 (9.55, 10.34)
*P*-trend^4^		0.003	0.04

Abbreviations: *AA* Arachidonic acid, *DGLA* Dihomo-γ-linolenic acid, *DHA* Docosahexaenoic acid, *DPA* Docosapentaenoic acid, *EPA* Eicosapentaenoic acid, *LA* Linoleic acid, *PUFA* Polyunsaturated fatty acid.

^1^Adjusted for age (y, continuous), sex and workplace [A (surveyed in July) or B (surveyed in November)].

^2^Adjusted for age (y, continuous), sex, workplace [A (surveyed in July) or B (surveyed in November)], smoking status (non-smoker or smoker), BMI (kg/m^2^, continuous), alcohol consumption (non-drinker, <20 g/d or ≥20 g/d), occupational physical activity (sedentary work or active work), non-occupational physical activity (0, 0 < to <5 or ≥5 MET-h/wk), serum folate (ng/mL, continuous), vitamin B6 intake (mg/1000 kcal, continuous) and vitamin B12 intake (μg/1000 kcal, continuous).

^3^Geometric means (95% confidence interval) of serum homocysteine (nmol/mL).

^4^P-trend values were based on multiple regression analysis, with ordinal numbers 0–2 assigned to tertile categories of each fatty acid.

^5^N-6 PUFA is the sum of LA, DGLA, and AA; n-3 PUFA is the sum of EPA, DPA, and DHA; PUFA is the sum of n-6 and n-3 PUFA.

The relation between serum CE fatty acid composition and homocysteine concentration is shown in Table 3. A significant inverse association was observed for total n-3 PUFA, ALA, EPA and DHA with adjustment for age, sex and workplace (P-trend = 0.01, 0.02, 0.02 and 0.02, respectively). However, these associations were attenuated in the fully adjusted model (P-trend = 0.34, 0.99, 0.40 and 0.34, respectively). Total n-6 PUFA and its components were not appreciably associated with homocysteine concentration.

**Table 3:** **Mean serum homocysteine concentrations according to tertile of n**-**3 PUFA and n**-**6 PUFA in serum CE**

**Fatty acid tertile**	**n**	**Model 1**^**1**^	**Model 2**^**2**^
PUFA			
<65.27%	166	10.51 (10.05, 10.99)^3^	10.40 (9.98, 10.83)
65.27–68.11%	165	10.20 (9.77, 10.65)	10.11 (9.73, 10.50)
≥68.12%	165	10.04 (9.61, 10.49)	10.25 (9.85, 10.66)
*P*-trend^4^		0.17	0.66
n-6 PUFA^5^			
<60.77%	166	10.27 (9.81, 10.75)	10.19 (9.77, 10.62)
60.77–64.19%	165	10.28 (9.85, 10.73)	10.27 (9.88, 10.66)
≥64.20%	165	10.20 (9.76, 10.68)	10.30 (9.89, 10.73)
*P*-trend^4^		0.86	0.74
LA (18:2n-6)			
<52.45%	166	10.20 (9.75, 10.68)	10.21 (9.79, 10.64)
52.45–56.90%	165	10.27 (9.84, 10.71)	10.23 (9.85, 10.63)
≥56.91%	165	10.28 (9.83, 10.75)	10.31 (9.90, 10.75)
*P*-trend^4^		0.83	0.74
GLA (18:3n-6)			
<0.44%	166	10.03 (9.61, 10.48)	10.13 (9.74, 10.53)
0.44–0.66%	165	10.27 (9.84, 10.72)	10.26 (9.88, 10.66)
≥0.67%	165	10.45 (10.01, 10.92)	10.36 (9.97, 10.77)
*P*-trend^4^		0.20	0.43
DGLA (20:3n-6)			
<0.45%	166	10.15 (9.71, 10.60)	10.15 (9.75, 10.57)
0.45–0.56%	165	10.50 (10.06, 10.96)	10.55 (10.16, 10.96)
≥0.57%	165	10.11 (9.67, 10.57)	10.05 (9.64, 10.48)
*P*-trend^4^		0.93	0.81
AA (20:4n-6)			
<6.06%	166	10.42 (9.98, 10.87)	10.34 (9.95, 10.74)
6.06–7.17%	165	10.20 (9.77, 10.65)	10.23 (9.85, 10.63)
≥7.18%	165	10.13 (9.71, 10.57)	10.18 (9.79, 10.58)
*P*-trend^4^		0.37	0.58
n-3 PUFA^5^			
<3.35%	166	10.73 (10.27, 11.21)	10.45 (10.04, 10.88)
3.35–4.71%	165	10.25 (9.82, 10.69)	10.14 (9.75, 10.54)
≥4.72%	165	9.79 (9.36, 10.23)	10.16 (9.36, 10.58)
*P*-trend^4^		0.01	0.34
ALA (18:3n-3)			
<0.56%	167	10.54 (10.10, 11.00)	10.25 (10.03, 10.48)
0.56–0.67%	166	10.47 (10.04, 10.92)	10.25 (10.03, 10.47)
≥0.68%	163	9.75 (9.33, 10.18)	10.25 (10.03, 10.48)
*P*-trend^4^		0.02	0.99
EPA (20:5n-3)			
<1.66%	166	10.72 (10.26, 11.20)	10.45 (10.03, 10.88)
1.66–2.79%	165	10.12 (9.70, 10.55)	10.12 (9.75, 10.52)
≥2.80%	165	9.93 (9.50, 10.38)	10.18 (9.77, 10.60)
*P*-trend^4^		0.02	0.40
DHA (22:6n-3)			
<1.00%	166	10.73 (10.27, 11.20)	10.49 (10.09, 10.91)
1.00–1.31%	165	10.09 (9.67, 10.53)	10.05 (9.68, 10.44)
≥1.32%	165	9.95 (9.52, 10.40)	10.21 (9.81, 10.63)
*P*-trend^4^		0.02	0.34

Abbreviations: *AA* Arachidonic acid, *ALA* A-linolenic acid, *CE* Cholesterol ester, *DGLA* Dihomo-γ-linolenic acid, *DHA* Docosahexaenoic acid, *EPA* Eicosapentaenoic acid, *GLA* γ-linolenic acid, *LA* Linoleic acid, *PUFA* Polyunsaturated fatty acid.

^1^Adjusted for age (y, continuous), sex and workplace [A (surveyed in July) or B (surveyed in November)].

^2^Adjusted for age (y, continuous), sex, workplace [A (surveyed in July) or B (surveyed in November)], smoking status (non-smoker or smoker), BMI (kg/m^2^, continuous), alcohol consumption (non-drinker, <20 g/d or ≥20 g/d), occupational physical activity (sedentary work or active work), non-occupational physical activity (0, 0 < to <5 or ≥5 MET-h/wk), serum folate (ng/mL, continuous), vitamin B6 intake (mg, continuous) and vitamin B12 intake (μg/1000 kcal, continuous).

^3^Geometric means (95% confidence interval) of serum homocysteine (nmol/mL).

^4^P-trend values were based on multiple regression analysis, with ordinal numbers 0–2 assigned to tertile categories of each fatty acid.

^5^N-6 PUFA is the sum of LA, GLA, DGLA, and AA; n-3 PUFA is the sum of ALA, EPA, and DHA; PUFA is the sum of n-6 and n-3 PUFA.

## Discussion

In this study among Japanese, we found an inverse association between DHA in serum phospholipids and homocysteine concentration. The other PUFAs were not significantly associated with homocysteine. This study is among few investigations on the association between fatty acid composition in serum CE or phospholipids and homocysteine concentration in a healthy population with high n-3 PUFA intakes.

The present finding of an inverse association between DHA in phospholipids and homocysteine was consistent with previous data [17-21]. In an Australian study of 139 healthy men [17], plasma homocysteine concentration was inversely associated with DHA in phospholipids after controlling for blood vitamin B12 and folate. In a Chinese study of middle-aged men including 128 omnivores and 103 vegetarians, an inverse association between DHA in phospholipids and homocysteine was observed with adjustment for blood vitamin B12 and folate [19]. In two other Chinese studies in 81 patients with hyperlipaemia [18] and 150 healthy subjects [20], plasma homocysteine concentrations were significantly, inversely correlated with DHA in phospholipids with adjustment for sex, age and BMI. In an Indian study among 49 patients with pre-eclampsia, plasma homocysteine concentrations were significantly, inversely correlated with DHA in the erythrocyte after adjusting for age, BMI and gestation [21]. The present, larger study with adjustment for three major B-vitamins (folate and vitamins B6 and B12) involved in homocysteine metabolism confirmed the association between blood levels of DHA and homocysteine, suggesting an independent role of DHA in the metabolism of homocysteine.

In the present study, homocysteine concentrations were significantly, inversely associated with EPA and ALA in serum CE and EPA in serum phospholipids in an age-, sex- and workplace-adjusted model. However, these associations were attenuated and no longer statistically significant after multivariate adjustment, largely due to confounding by serum folate. This observation is compatible with null finding in previous studies that measured fatty acid composition in blood [18-20]. Similar to our study, a Chinese study showed that plasma EPA levels were not statistically significantly associated with homocysteine after adjustment for folate [19]. In an experimental study [12], EPA and ALA showed a smaller effect of down-regulation on methionine adenosyltransferase (MAT), which is involved in homocysteine metabolism, than DHA. EPA and ALA may have a minor role, if any, in the determination of homocysteine concentrations.

N-6 PUFAs were not associated with serum homocysteine concentration in the present study. Few studies examined the associations between blood n-6 PUFA levels and homocysteine concentrations [19,20,24]. Plasma phospholipid n-6 PUFA was significantly positively associated with plasma homocysteine in vegetarians [19], diabetes patients [20] and general population [24]. In one of these studies, however, n-6 PUFA was not associated with plasma homocysteine in omnivores [19]. Given limited epidemiologic evidence and lack of plausible biological mechanism supporting a role of n-6 PUFAs in homocysteine metabolism, more experimental and human studies are required on this issue.

In the present study, homocysteine concentrations were significantly associated with phospholipid DHA but not with CE DHA, a finding for which we have no plausible explanation. However, this might be attributable to the fact that CE DHA had a much lower mean and smaller variation than did phospholipid DHA (mean, 1.2% versus 7.6%; SD, 0.4% versus 1.7%). Due to such a small proportion of DHA in CE, it would be difficult to distinguish persons in high DHA status from those in low status with data on serum CE fatty acids, leading to a low probability of detecting an association with homocysteine, if any.

The mechanism underlying the association between n-3 PUFAs and homocysteine is not yet fully understood. Homocysteine concentrations are partly determined by genetic factors, including genes encoding enzymes involved in homocysteine metabolism such as MAT, cystathionine-γ-lyase (CSE) and 5-methyltetrahydrofolate reductase (MTHFR) [12]. In an experimental study using human HepG2 cell, administration of n-3 PUFA up-regulated CSE and MTHFR mRNA expressions and down-regulated MAT mRNA expression [12]. Compared with EPA and ALA, DHA showed more pronounced effects in down-regulating MAT mRNA expression [12]. Such regulatory effect of n-3 PUFA on expression of these genes may lead to decreased homocysteine concentration.

Major strengths of this study include a high participation rate (91%) in a well-defined working population, the use of a biomarker of dietary fatty acid intake and adjustment for potentially important confounders including smoking, physical activity, vitamin B6, vitamin B12 and folate. In the present population, serum folate levels were significantly associated with both serum n-3 and n-6 PUFA and homocysteine levels, and some statistically significant associations in a folate-unadjusted model disappeared after adding serum folate to the model, suggesting the importance of adjustment for blood folate as a confounder. However, this study has some limitations. First, because of its cross-sectional design, we cannot infer causality from our finding. Second, we measured serum fatty acid composition and homocysteine only at one point in time, which may not reflect long-term status. Third, due to close inter-correlations among PUFAs, the observed association for a specific fatty acid may reflect its joint effect with other fatty acids on homocysteine. Fourth, we stored serum samples at −20°C until fatty acid measurement. In this condition, there might be selective loss of PUFA due to the oxidation over time ([30]). Fifth, intra-assay variation for DHA measurement was relatively large compared with other fatty acids. This measurement error is probably non-differential and might have attenuated the DHA – homocysteine association. Sixth, although we found a statistically significant association between serum homocysteine levels and DHA, the difference in mean homocysteine level between the highest and lowest tertiles of DHA was small (0.63 nmol/mL). It remains elusive whether such a difference is of clinical significance. Seventh, although we have adjusted for known and suggested factors associated with homocysteine levels, we cannot exclude a possibility of bias due to other confounders or residual confounding. For instance, we adjusted for dietary vitamin B6 and B12 intakes, but not for their serum levels due to a lack of these data. The adjustment for blood levels of these vitamins may provide different results. Finally, the study subjects did not represent a random sample of the Japanese population and thus caution is required in generalizing the present findings.

## Conclusions

Lower serum homocysteine concentrations were observed among those with higher DHA in serum phospholipids in an apparently healthy Japanese population. This finding adds to evidence for a role of DHA in lowering homocysteine concentrations at relatively high DHA levels. The protective effect of n-3 PUFA may not be limited to CHD; evidence from observational studies support a role of n-3 PUFA against dementia [31], the risk of which increased among persons with high homocysteine levels [32]. Prospective studies in populations with high fish consumption are required to clarify whether a higher DHA status at baseline is associated with a future decline in blood homocysteine levels and risk of homocysteine-related diseases.

## Competing interests

The authors declare that they have no competing interests.

## Authors’ contribution

AK analyzed data and drafted the manuscript. KK (Kurotani), NMP, AN, and KK (Kuwahara) helped to analyze data and draft the manuscript. MS and YE measured serum fatty acid composition and participated in the revision of the manuscript. TM conceived the study, and participated in its design and coordination and helped to draft the manuscript. All authors read and approved the final manuscript.
